# Immune-Checkpoint-Inhibitor-Related Lung Toxicity: A Multicentre Real-Life Retrospective Portrait from Six Italian Centres

**DOI:** 10.3390/life12081149

**Published:** 2022-07-29

**Authors:** Paolo Cameli, Paola Faverio, Katia Ferrari, Viola Bonti, Stefania Marsili, Maria Antonietta Mazzei, Francesca Mazzoni, Maurizio Bartolucci, Vieri Scotti, Federica Bertolini, Fausto Barbieri, Cinzia Baldessari, Chiara Veronese, Roberto Boffi, Matteo Brighenti, Diego Cortinovis, Massimo Dominici, Alberto Pesci, Elena Bargagli, Fabrizio Luppi

**Affiliations:** 1 Respiratory Diseases Unit, Department of Medicine, Surgery and Neurosciences, Siena University Hospital, University of Siena, 53100 Siena, Italy; bargagli2@unisi.it; 2 Respiratory Unit, University of Milan-Bicocca, S. Gerardo Hospital, 20900 Monza, Italy; paola.faverio@unimib.it (P.F.); alberto.pesci@unimib.it (A.P.); fabrizio.luppi@unimib.it (F.L.); 3 Respiratory Medicine Unit, Careggi University Hospital, 50134 Florence, Italy; ferrarik@aou-careggi.toscana.it (K.F.); bontiv@aou-careggi.toscana.it (V.B.); 4 Oncology Unit, Azienda Ospedaliera Universitaria Senese, 53100 Siena, Italy; s.marsili@ao-siena.toscana.it; 5 Department of Medical, Surgical and Neuro Sciences, University of Siena, 53100 Siena, Italy; mariaantonietta.mazzei@unisi.it; 6 Unit of Diagnostic Imaging, Department of Radiological Sciences, Siena University Hospital, Viale Bracci 10, 53100 Siena, Italy; 7 Medical Oncology Unit, Careggi University Hospital, 50134 Florence, Italy; mazzonif@aou-careggi.toscana.it; 8 Radiology Department, Careggi University Hospital, 50134 Florence, Italy; bartoluccim@aou-careggi.toscana.it; 9 Radiation Oncology Unit, Careggi University Hospital, 50134 Florence, Italy; vieri.scotti@unifi.it; 10 Division of Medical Oncology, Modena University Hospital, 41100 Modena, Italy; bertolini.federica@policlinico.mo.it (F.B.); barbieri.fausto@policlinico.mo.it (F.B.); cinzia.baldessari@libero.it (C.B.); massimo.dominici@unimore.it (M.D.); 11 SS Lung Unit, Critical Care Medicine Department, Foundation IRCCS National Cancer Institute of Milan, 20133 Milan, Italy; chiara.veronese@istitutotumori.mi.it (C.V.); roberto.boffi@istitutotumori.mi.it (R.B.); 12 Medical Oncology, ASST Cremona, 26100 Cremona, Italy; m.brighenti@asst-cremona.it; 13 SC Medical Oncology, SS Lung Unit, ASST H S Gerardo, 20900 Monza, Italy; d.cortinovis@asst-monza.it

**Keywords:** nivolumab, pulmonary fibrosis, PD-1 inhibitors, lung cancer, checkpoint inhibitor toxicity

## Abstract

Background: Immune checkpoint inhibitors (ICIs) have revolutionized the therapeutic horizons of various cancers. However, immune-related adverse events have been reported, including interstitial lung diseases. Our aim was to describe the clinical and radiological features and survival of a multicentre cohort of patients who developed ICI-related lung toxicity. Methods: Six Italian centres were involved in the study. Patients who were treated with anti-PD-1/PD-L1 and CTLA-4 mAbs and developed ICI-related lung toxicity were recruited retrospectively to study clinical, radiological, immunological and survival data. Results: A total of 41 patients (25 males, 66.8 ± 9.9 years) were enrolled. Lung toxicity occurred after 204.3 ± 208.3 days of therapy, with ground glass opacities being the most common HRCT pattern (23 cases). Male sex, lung cancer and acute respiratory failure were associated with a shorter latency of toxicity (*p* = 0.0030, *p* = 0.0245 and *p* = 0.0390, respectively). Patients who required high-flow oxygen therapy showed significantly worse survival (*p* = 0.0028). Conclusions: Our cohort showed heterogeneous clinical and radiological aspects of ICI-related lung toxicity, with a latency not limited to the first year of treatment. Severity was mainly mild to moderate, although life-threatening events did occur. Our data indicate that strict long-term follow-up is needed to enable early diagnosis and appropriate management.

## 1. Introduction

Interstitial lung diseases (ILDs) are characterized by acute and chronic bilateral parenchymal infiltration due to known and unknown causes. Those due to drug toxicity typically have acute presentation with exertional dyspnea and abnormal gas exchange until manifestation of acute respiratory failure. Drug-induced ILD is associated with various radiological patterns, including usual interstitial pneumonia (UIP), non-specific interstitial pneumonia (NSIP), diffuse alveolar damage, organizing pneumonia (OP), hypersensitivity pneumonitis (HP) and acute interstitial pneumonia detected by high-resolution computed tomography (HRCT) of the chest [1]. Standing the clinical non-specificity and the radiological heterogeneity, diagnosis of drug-induced ILD may be very challenging, and a detailed collection of medical history and of the timeline of patient’s drug exposition is crucial for an optimal diagnostic assessment of ILD subjects, as stated by the international guidelines for diagnosis of idiopathic pulmonary fibrosis (IPF) [2].

In the new era of the antitumor drug growth, ILD associated with antineoplastic drugs has been reported. Interstitial lung toxicity was recently described in patients treated with anti-neoplastic agents that target molecular pathways, including epidermal growth factor receptor tyrosine kinase inhibitors (EGFR-TKIs) and mammalian target of rapamycin (mTOR) inhibitors, particularly if associated with chemotherapy or radiotherapy [3,4,5].

Immune checkpoint inhibitors (ICIs) with particular activity against the PD-1/PD-L1 axis and CTLA-4 are now a widely used therapy for many solid tumours [6]. These inhibitors, including nivolumab and pembrolizumab, are normally tolerated much better than chemotherapy; however, a relevant number of adverse events (so-called immune-related adverse events (irAE)), due to immune system stimulation and reduction in self-tolerance mechanisms elicited by anti-PD-1/PD-L1 agents, have begun to emerge. Among irAEs, forms of lung toxicity are challenging not only for their potential life-threatening nature but also because their signs and symptoms may overlap with those of disease progression or intercurrent lung infections, causing a relevant probability of misdiagnosis and eventually leading to a delay in a proper therapeutic management. Moreover, the immunopathological pathways underlying ICI-related respiratory complications are still far from being fully understood, as well as the pathobiological mechanisms for which patients may explicit lung toxicity with very heterogenous degrees of extension, severity and radiological patterns. Consequently, reliable biomarkers for differential diagnoses are lacking, and we are currently not able to predict with an acceptable accuracy the individual risk of experiencing lung toxicity and relative severity during ICI treatment.

The real incidence and features of lung toxicity due to ICIs in patients with lung cancer have been evaluated in very few studies [7]. The incidence of ICI-induced pneumonitis is reported to vary in the range of 1–12%, reflecting diagnostic and classification difficulties [8,9]. A meta-analysis of 20 studies showed an incidence of severe pulmonary toxicities in 0.8% of the population, while the overall incidence was 2.7% [9]. The incidence of pneumonitis varied with different ICI regimens: anti-PD-L1 is associated with a lower probability of lung toxicity than anti-PD-1, while a combined regimen of anti-PD-1/PDL-1 + anti-CTLA4 seems associated with a higher probability of irAEs [8,10,11,12].

Compared to the lung toxicity of chemotherapy, ICI-related pneumonitis seems to determine a steroid-sensitive ILD with a better prognosis, although fatal cases have also been described [13,14,15]. Kato et al. showed that pre-existing inflammatory lung conditions, including radiation pneumonitis and bacterial pneumonia, may be risk factors for the development of lung toxicity in patients exposed to ICIs, probably due to an immune-mediated adverse reaction [16]. Moreover, Kanai et al. showed that the incidence of nivolumab-related pneumonitis was greater in patients with pre-existing ILD [17].

The aim of the present study was to describe the features of a multicentric population of patients who developed an ILD during treatment with ICIs. Particular attention has been paid to evaluate the clinical and radiological characteristics of these patients and their specific prognostic impact in terms of survival and response to treatment.

## 2. Materials and Methods

### 2.1. Study Design

In this multicentric study, we retrospectively reviewed the clinical records and radiological images of patients treated with ICIs who developed lung toxicity in the period from August 2015 to January 2019. Patients were enrolled from six Italian hospitals, including Siena, Florence, Modena, Monza, Cremona and Milan pulmonology and oncology units. Demographic, clinical, histopathological, radiological and therapeutic data were collected and entered in an electronic database shared by all researchers. The type and duration of ICI treatment were recorded in detail, as well as lung-toxicity-related symptoms and severity. Clinical status was evaluated through patient follow-up from diagnosis of lung toxicity to clinical outcome or 1 July 2019.

Experienced radiologists classified chest high-resolution computed tomography (HRCT) features together with pulmonologists, radiotherapists and oncologists during multidisciplinary discussion, according to international guidelines [18]. Radiologists were asked to define the main CT patterns focusing on interstitial abnormalities, including ground glass opacities (GGO), lung fibrosis, OP and HP-like parenchymal features. Presence/absence of emphysema and pleural effusion were recorded as well.

Grading of lung toxicity was assessed according to American Society of Clinical Oncology (ASCO) guidelines [18] and Common Terminology Criteria for Adverse Events (CTCAE) (version 4.0) [19], while the therapeutic management of lung toxicity was discussed case by case through multidisciplinary discussion, following the European Society for Medical Oncology (ESMO) guidelines [20].

If available, pulmonary function test (PFT) parameters, including the analysis of alveolar diffusion lung capacity, were recorded. To be included in the analysis, PFTs have to be performed during the diagnostic assessment of ICI-related lung toxicity and before the start of any pharmacological treatment.

Microbiological and lymphocyte population data were collected when bronchoalveolar lavage (BAL) results were available.

The study was approved by the local ethical committee of every centre. The study was conducted according to Declaration of Helsinki principles.

### 2.2. Pulmonary Function Tests

The following lung function measurements were recorded according to American Thoracic Society (European Respiratory Society (ATS/ERS) standards [21,22], using a plethysmograph with corrections for temperature and barometric pressure: forced expiratory volume in the first second (FEV1), forced vital capacity (FVC), FEV1/FVC ratio, total lung capacity (TLC), residual volume (RV), carbon monoxide lung transfer factor (TLCO) and capacity carbon monoxide lung transfer factor/alveolar volume (TLCO/VA). These measurements were collected in patients able to perform properly lung function tests.

### 2.3. High-Resolution Computed Tomography

Chest HRCT was performed with a 64-row CT scanner. All patients were imaged in supine position, with breath-holding at maximum lung capacity. HRCT examination was obtained in axial scanning mode for 12 patients and in spiral scanning mode for 29 patients. Imaging reconstruction was performed through “bone plus” and standard algorithms.

### 2.4. Bronchoalveolar Lavage

Bronchoscopy with bronchoalveolar lavage was performed for diagnostic purposes according to ATS/ERS Guidelines on BAL [23]. BAL fluid was obtained by instillation of four 60 mL aliquots of saline solution by a fibrobronchoscope wedged in the subsegmental bronchus of the target lobe according to HRCT scan. In case of diffuse and bilateral lung involvement, BAL sampling was performed in the middle lobe or in the lingula, according to the operator’s judgement. All BAL sampling collections were performed for microbiological and cytological examination. Cellular analysis of BAL was performed in 5 subjects (Siena Centre): the first sample was kept separate from the others and was not used for immunological tests. BAL was filtered through sterile gauze. Cellularity was determined by cytocentrifuging glass slides for 5 min at 40× *g*. Staining was performed with a May Grunwald Giemsa stain kit (DiaPath, Italy, Europe). At least 500 cells were counted. Cell viability was determined by trypan blue exclusion in a Burker chamber.

### 2.5. Statistical Analysis

Data were expressed as mean ± standard deviation. Normal distribution was assessed through the Kolmogorov–Smirnov test. Since our cohort did not show normal distribution of variables, differences between groups were calculated using non-parametric tests (Mann–Whitney or Kruskal–Wallis test, as appropriate). Unadjusted survival analysis was obtained using Kaplan–Meier curves. Time-to-event endpoints were compared using a two-sided log-rank test. A *p* value ≤ 0.05 was considered significant.

Stata version 15.1 (Stata corp, College Station, TX, USA), Microsoft Excel (Redmond, WA, USA) and Graphpad Prism 5.0 (San Diego, CA, USA) for Windows were used for statistical analysis and to plot the figures.

## 3. Results

The overall study population was composed of 41 patients (25 males, 66.8 ± 9.9 years) with different types of cancer (lung, breast, melanoma, mesothelioma and nasopharynx), the majority with lung cancer, who reported lung toxicity during treatment with PD-1, PDL-1 and CTLA-4 inhibitors. Demographic features, smoking status, treatment data, respiratory functional parameters (when available) and clinical symptoms at diagnosis of lung toxicity are summarized in Table 1.

Our population included 32 patients with non-small-cell lung cancer (19 adenocarcinoma and 13 squamous cell carcinoma), 2 with melanoma, 2 with breast cancer, 2 with kidney cancer, 1 with pleural mesothelioma, 1 with appendix cancer and 1 with nasopharyngeal cancer. The population was predominantly composed of current or former smokers and were symptomatic in the majority of cases. Hospitalization for diagnostic assessment and treatment was requested in 35 patients (85%).

PFT data were only available for 15 patients, who on average showed mild mixed impairment of lung volumes associated with a moderate reduction in TLCO.

BAL data were available for 10/41 patients; microbiological examination of all sputum and BAL samples was negative. Among the 31 patients without BAL samplings, 9 patients were judged as too clinically unstable to undergo the procedure, 6 patients refused to give consent to endoscopic exam while 16 cases were labelled with a high confidence provisional diagnosis of lung toxicity according to clinical symptoms, laboratory exams, timeline of drug exposure and radiological CT pattern. BAL cellular analysis was performed only in five patients belonging to the Siena Centre, showing, on average, a higher percentage of lymphocytes (25.7%) and neutrophils (10.2%), counteracted by lower levels of macrophages (55.1%).

**Figure 1:**
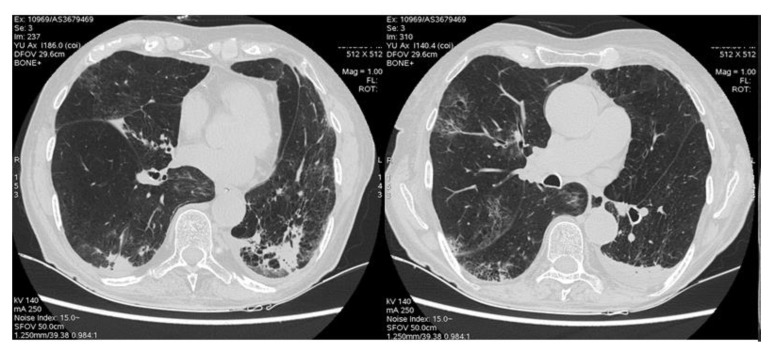
CT features of one patient diagnosed with nivolumab-related drug toxicity in the Siena Centre. Bilateral peripheral parenchymal consolidations involved lower lobes, associated with patchy GGO in the middle lobe and lower lobes and left pleural effusion. The patient was hospitalized due to dyspnea and chest pain.

ICIs were used as first-, second- or third-line therapy in 8, 28 and 5 patients, respectively. Only one patient, who had melanoma, was treated with a combination of nivolumab and ipilimumab. Radiotherapy was performed in 19 patients (16 with lung cancer, 1 with breast cancer, 1 with mesothelioma and 1 with nasopharyngeal cancer). No patient showed chest HRCT evidence of ILD before starting chemotherapy or radiotherapy. Table 2 shows HRCT features, with GGO and HP-like parenchymal features being the predominant patterns (Figure 1).

**Figure 2:**
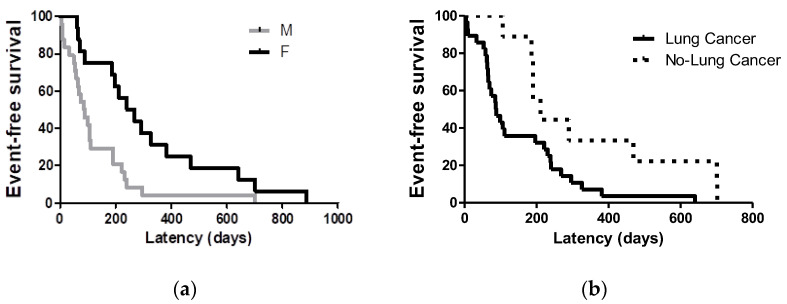
Kaplan–Meier curves for latency time according to: (**a**) sex and (**b**) type of cancer. Male gender and lung cancer were significantly associated with a worse survival after lung toxicity onset (*p* = 0.0030 and *p* = 0.0245, respectively).

Lung toxicity occurred after 204.3 ± 208.3 days of therapy (first-line therapy: 200.5 ± 230.9 days; second-line therapy: 209.1 ± 205.2 days; third-line therapy: 184.4 ± 257.5 days; *p* = 0.8325). In our cohort, male sex and lung cancer were associated with a significantly shorter latency of lung toxicity (*p* = 0.0030 and *p* = 0.0245, respectively) (Figure 2).

Concerning radiological pattern, patients with cryptogenic organizing pneumonia showed a longer latency than those with GGO and HP-like parenchymal features, though the difference was not statistically significant (*p* = 0.0827). No differences in latency were found in relation to age, smoking status, diagnosis of COPD, HRCT evidence of emphysema, prior radiotherapy, prior chemotherapy or ICI regimen.

After diagnosis of lung toxicity, all but seven patients underwent steroid treatment (dosage ranged from 8 mg/day to 2 mg/kg/day, on the basis of severity of disease). Patients experiencing acute respiratory failure and with OP pattern at CT scan were treated with a significantly higher steroid daily dose than those without need for oxygen therapy and other radiological features (*p* = 0.00256 and *p* = 0.0347, respectively). PD-1 inhibitor therapy was temporarily or permanently discontinued in 7 and 22 patients, respectively, according to ASCO and CTCAE v.4.0 indications (17,18).

**Figure 3:**
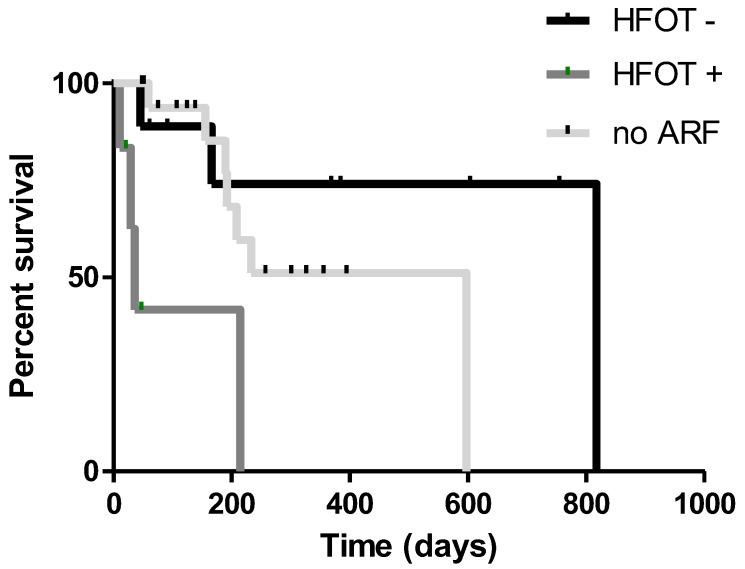
Kaplan–Meier curves for overall survival according to acute respiratory failure development and relative need for high-flow oxygen therapy. Patients with more severe respiratory impairment needing high-flow oxygen therapy showed the worst survival. ARF: acute respiratory failure; HFOT: high-flow oxygen therapy.

**Figure 4:**
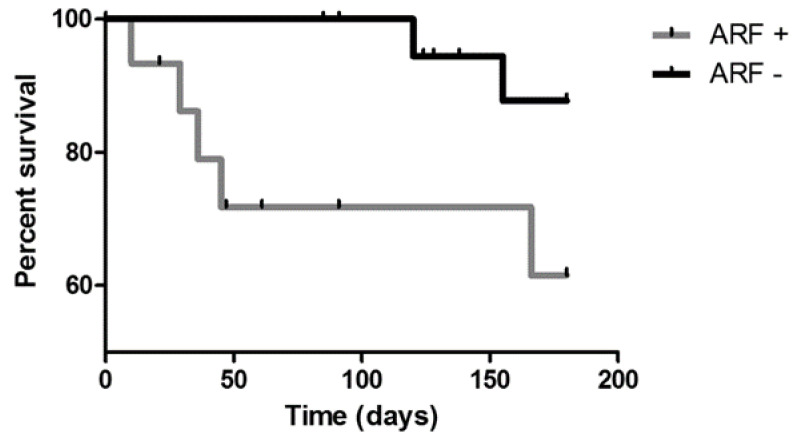
Kaplan–Meier curves for 6 mo percent survival according to acute respiratory failure development. A significant increase in mortality was observed in patients experiencing acute respiratory failure (*p* = 0.0454). ARF: acute respiratory failure.

Fifteen patients (36.5%) experienced acute respiratory failure: six suffered acute deterioration of clinical status and required high-flow nasal cannula oxygen therapy to support respiratory function. These patients had a worse prognosis than other patients (*p* = 0.0028) (Figure 3). GGO and fibrotic interstitial abnormalities were more frequently reported in patients experiencing respiratory failure than subjects without need for oxygen therapy (*p* = 0.0151 and *p* = 0.0454, respectively). Moreover, acute respiratory failure was associated with a significantly shorter latency of lung toxicity onset (*p* = 0.0390) and a higher six-month mortality rate (*p* = 0.0454) (Figure 4).

Regarding survival analysis, in the entire population, 15 patients died 181.5 ± 12.5 days after diagnosis of lung toxicity (median survival 155 days). Six deaths occurred within 3 months of onset of lung toxicity (five males). These patients were all current or former smokers and had a mean age of 68 ± 13 years; 50% of them had undergone radiotherapy before starting ICI and only one had received PD-1 inhibitor as first-line therapy. This subgroup did not show any significant differences in demographic features, smoking status, type of cancer or ICI regimen with respect to patients who died of cancer. In this subgroup, two patients underwent BAL, and their microbiological tests were negative. HRCT scans showed no evidence of cancer progression: bilateral reticular abnormalities, OP pattern and bilateral GGO were found in two, one and four patients, respectively.

## 4. Discussion

The present study summarizes the experience of six Italian centres in the diagnosis and management of ICI-related lung toxicity. Several drugs may induce lung toxicity, and an association with anti-PD-1/PDL-1 and CTLA-4 ICIs has been recently reported [7,24,25,26]. Certain clinical factors may favour the onset of ICI pneumonitis: cancer subtype (lung > melanoma) [9] and exposure to tobacco smoke, whereas COPD does not seem to influence the incidence of lung toxicity [12]. This variability of incidence among solid tumours suggests that some pre-existing conditions such as smoke exposure added to neoplastic lung involvement may alter the microenvironment, giving a differential risk to develop this irAE.

Our study partially confirmed these indications, as most of our patients were affected with lung cancer. Consequently, a male prevalence and a high percentage of current or former smokers were found, as expected. Male patients also showed a shorter latency than females between treatment/drug exposure and onset of lung toxicity, irrespective of age, smoking status or comorbidities. The latency observed in our cohort was in line with the findings of previous studies, despite a very high standard deviation [8,11]. The dispersion of our data may be due to the different patterns of interstitial lung toxicity observed in our relatively small cohort. The main radiological patterns were OP, HP-like parenchymal features and GGO, in line with previous data [8,27,28]; however, other radiological interstitial features were observed in these patients, including NSIP and UIP patterns. In our study, fibrotic ILD developed in a small percentage of patients, with a longer, albeit not significant, latency with respect to GGO and HP-like parenchymal features. Due to the retrospective nature of the study, the potential causative role of immunotherapy in inducing ILD cannot be accurately assessed. However, on this topic, Pneumotox lists a wide range of forms of lung toxicity, including fibrotic ILDs, associated with these drugs [29]. Moreover, the recent Fleischner Society paper also underlined that ICI-related lung toxicity is heterogeneous in terms of clinical onset, course and radiological features and reported as more common the same CT patterns described in our study [30]. Therefore, our findings implicitly confirm these indications, showing a great variability in latency, type and severity of lung involvement, and suggest that long-term monitoring of these patients for the development of ILD could be useful for earlier detection and treatment.

Concerning the prognostic significance of different CT patterns, to our knowledge, only a paper by Watanabe et al. [28] reported a direct comparison, demonstrating that patients developing GGO were affected by a worse prognosis and life expectancy than in those with OP. Our data seem to be in line with these observations, as the majority of patients who died early showed chest HRCT evidence of the GGO pattern. This finding may be explained by the different clinical onset and severity associated to specific CT patterns: compared with OP or other ILD patterns, the presence of diffuse and bilateral GGO is typically associated with an acute/hyperacute clinical deterioration, frequently combined with a potentially life-threatening hypoxemic respiratory failure, mimicking a condition of acute respiratory distress syndrome (ARDS).

Fatal ICI-related lung toxicity has rarely been reported in the literature [12]: our study contributed to the topic by reporting the cases of six patients who died of acute lung toxicity 3 months after beginning immunotherapy. Unfortunately, no predictive biomolecular markers are available to accurately forecast the development of lung side effects with ICIs, and we failed to find specific features associated with radiological patterns or the severity of these episodes. We observed that acute respiratory failure, particularly cases requiring high-flow oxygen therapy, is associated with worse early-term prognosis and therefore needs to be rapidly assessed to rule out lung toxicity.

Concerning clinical presentation, cough and dyspnea were the most common symptoms, while fever was only described in four patients. The literature also shows that the incidence of fever varies widely [31]. Fever may incorrectly suggest that respiratory symptoms are due to an infection, delaying diagnosis of lung toxicity. As some patients were asymptomatic at diagnosis and symptoms were non-specific, PFTs, including DLCO, may be proposed as a baseline evaluation, also since a reduction in DLCO was found in all our patients who performed PFTs. No data are available on the predictive role of lung function and DLCO monitoring for early detection of patients suspected to have lung toxicity. Therefore, PFTs and DLCO evaluation may be proposed as a non-invasive screening and monitoring tool before and during treatment.

Little data on BAL cell phenotyping in these patients are available in the literature; a recent paper by Delaunay et al. found a predominance of lymphocytes, as expected, evidence sustaining an immune-mediated mechanism of ICI-related ILD [32]. Even if limited by small numerosity, our findings confirmed previous data showing a significant increase in lymphocytes in BAL samplings. The increase of the lymphocytic subpopulation in BAL is probably related to an immunoinflammatory imbalance induced by ICIs, leading to a predominance of proinflammatory factors in lung tissue. It is still largely unknown why ICI-related lung toxicity may show up with different radiological patterns, as reported in literature and confirmed by our findings. Study of BAL cells (especially lymphocyte and eosinophil percentages) may therefore not only be useful to exclude infections but also to phenotype immunological lung involvement secondary to drug exposure, helping to optimize diagnosis and consequently therapeutic management. Thus, we believe that BAL can be useful and should be performed whenever possible.

Our study has several weaknesses: due to its retrospective nature, the risk of referral and reporting bias is significant, despite the considerable experience of these specialist centres. The number of cases of lung toxicity could be underestimated, as we only analysed major severe events. The real-world setting of the study implied a heterogeneous cohort in terms of type of cancer and treatment protocols, as well as the clinical management of every centre. The limited number of PFTs and BAL in our cohort is another limitation, since the ESMO guidelines strongly suggests, if possible, to perform bronchoscopy to rule out infection, and PFTs could represent a valid non-invasive method to address severity of lung impairment. The potential synergistic effect of chemo- or radiotherapy in inducing lung toxicity should not be underestimated but cannot be properly assessed using the present study design. This paper stimulates the necessity to improve the knowledge about this peculiar toxicity and underlines the importance of having a multidisciplinary team trained specifically to intercept and manage all immune-related adverse events as early as possible. This task force, permanently constituted in all large volume hospitals, may lead to minimize the mortality risk of all the patient developing ICI inducted pneumonitis. However, our experience describes the pattern of pulmonary toxicities and their management in a real-world practice in hospitals treating a large volume of cancer patients with ICIs.

## 5. Conclusions

Here we described the characteristics of a population of patients with cancer, who developed lung toxicity after treatment with ICIs. Although these drugs are generally safe, as suggested by the small cohort collected in six centres, we underline the need for a multicentre prospective study to evaluate the role of respiratory function screening and monitoring to improve prevention and early diagnosis of these potentially severe adverse effects.

## Figures and Tables

**Table 1:** Demographic and therapeutical features, functional parameters, clinical onset and grading of lung toxicity (according to ASCO guidelines) in our study population.

Study Population	
N°	41
Male (%)	25 (60.9)
Age (yrs)	66.8 ± 9.9
BMI (kg/m^2^)	26.6 ± 4.6
**Smoking history (pack-years)**	31.2 ± 24.6
Current: number (%)	8 (19.5)
Former: number (%)	26 (63.4)
Never: number (%)	7 (17.1)
**Clinical onset**	
Asymptomatic (%)	13 (31.7)
Cough (%)	8 (19.5)
Dyspnea (%)	22 (53.6)
Fever (%)	4 (9.7)
Respiratory failure (%)	15 (36.5)
**Grading Lung toxicity**	
1 (%)	14 (34.1)
2 (%)	12 (29.2)
3 (%)	9 (21.9)
4 (%)	6 (14.6)
**PFTs (N° patients)**	15
FVC l (% predicted value)	2.2 ± 0.6 (80.5 ± 17.9)
FEV1 l (% predicted value)	1.7 ± 0.6 (75.3 ± 23.9)
FEV1/FVC	71.5 ± 12.6
DLCO (% predicted value)	52.7 ± 15.4
**Therapy status**	
Nivolumab—1st line (%)	2 (4.8)
Nivolumab—2nd line (%)	25 (60.9)
Nivolumab—3rd line (%)	5 (12.2)
Pembrolizumab—1st line (%)	5 (12.2)
Pembrolizumab—2nd line (%)	3 (7.3)
Nivolumab + ipilimumab—1st line (%)	1 (2.4)

**Table 2:** CT features in our study population.

Predominant CT Patterns	
Organizing pneumonia (%)	6 (14.6)
Ground glass opacities (%)	23 (56)
HP-like parenchymal features (%)	8 (19.5)
Lung fibrosis (%)	4 (9.7)
Emphysema (%)	14 (34.1)
Pleural effusion (%)	3 (7.3)

## Data Availability

Data are contained within the article.
